# Exploring the Role of the Food Environment in Dietary Acculturation: A Study amongst Moroccan Immigrants in The Netherlands

**DOI:** 10.3390/ijerph18073328

**Published:** 2021-03-24

**Authors:** Jillian O’Mara, Wilma Waterlander, Mary Nicolaou

**Affiliations:** Department of Public and Occupational Health, Amsterdam Public Health Research Institute, Amsterdam UMC, University of Amsterdam, Meibergdreef 9, 1105 AZ Amsterdam, The Netherlands; w.waterlander@amsterdamumc.nl (W.W.); m.nicolaou@amsterdamumc.nl (M.N.)

**Keywords:** food environment, dietary acculturation, dietary patterns

## Abstract

Food environments play a role in immigrants’ dietary acculturation, but little is known about the directionality of the relationship. The objective was to explore the interaction between the food environment and food procurement behaviors in the process of dietary acculturation. A qualitative study design using in-depth interviews and a mapping exercise was conducted. The immigrant group studied used a variety of factors to select which foods to procure. Traditional foods were readily available, shifting the determining factors to a combination of affordability, acceptability and accessibility. The food environment is dynamic and responds to shifting market demands. Policies regarding food procurement behaviors should consider these upstream effects and be aware of the availability of traditional foods for immigrant groups.

## 1. Introduction

The dietary patterns of migrant-origin groups often differ from that of host populations [1] although there is diversity in the extent to which these differences persist given residence duration and contact with host cultures. Some groups are more likely to preserve traditional eating patterns, while other groups adopt the eating patterns of the host country more readily. These changing eating patterns are part of the process of dietary acculturation which can be described as a dynamic phenomenon that continues to evolve throughout the immigrant’s experience in the host country [2]. Changes in dietary behavior due to migration and, presumably, acculturation are likely to have an impact on health. Diet-related chronic diseases, such as obesity, diabetes and cardiovascular diseases have been shown to be more prevalent in some migrant-origin groups [3,4,5]. Understanding the determinants of dietary acculturation is necessary for the development of public health strategies to mitigate the unequal burden of chronic disease of migrant-origin groups.

The food environment, defined as “the collective physical, economic, policy and sociocultural surroundings, opportunities and conditions that influence people’s food and beverage choices and nutritional status,” [6], has been shown to play a role in dietary decision-making in the general population [7,8,9] and in the process of dietary acculturation of migrants [10]. Furthermore, food environments have been shown to negatively impact health by promoting increased unhealthy eating and decreased physical activity [11].

However, the role of the food environment within the context of the immigrant dietary experience, specifically in dietary acculturation, has not been extensively researched. In their model of dietary acculturation, Satia et al. [2] acknowledge the importance of the food environment for immigrant populations in food procurement. Many studies have corroborated that aspects of the food environment, such as availability of traditional foods, play an important role in the immigrant’s decision-making behavior [12,13,14]. In a study of immigrants in Ontario, Canada Rodriguez et al. [15] found the most important drivers of food purchasing behavior were access and affordability of both traditional and host country foods. However, these studies have not been explicit about which aspects of the food environment influence food procurement and, ultimately, acculturation, nor do they consider the dynamic nature of that relationship, i.e., the influx of immigrants may also shift the food environment in which they are living, providing more availability of traditional items over time and creating a bi-directional relationship.

In this study, we focus on people of Moroccan origin in Amsterdam, the Netherlands. Foreign immigration to the Netherlands has been ongoing since the 1960s and continues to increase, with Amsterdam at the epicenter. People of Moroccan origin first immigrated to Amsterdam in the 1960s when the Dutch offered low-paying jobs and made it possible for immigrants to get working visas [16]. In the 1990s, this shifted to primarily family reunification migration, and, in more recent years, the reason for migration has been cited as “work and study” [17]. People of Moroccan origin now make up the largest ethnic minority group, accounting for almost 10% of the population [18]. They show higher rates of obesity and type 2 diabetes than the native Dutch population [19], and there is evidence that obesity is an important contributor to cardiovascular disease risk in Moroccan women in particular [20]. In the last 50–60 years, people of Moroccan origin in Amsterdam have experienced the process of dietary acculturation and they have also impacted the food environment in this timeframe, making them a suitable group in which to study the dynamic interaction between migrants and the food environment.

Therefore, the objective of this study was to explore the interaction between the food environment and food procurement behaviors in the process of dietary acculturation by investigating how an immigrant group, people of Moroccan origin in Amsterdam, interact with the food environment for the procurement of food.

## 2. Methodology

### 2.1. Research Design

We used an explorative, qualitative study design to examine the opinions and subjective accounts [21] of the interviewees’ perceptions about their food environments and food purchasing behaviors.

### 2.2. Study Setting

Two neighborhoods, Amsterdam East and Amsterdam New-West, were selected as two different physical food environments based on their population of Moroccan immigrants, their separate geographical location and their different observed food environments. Moroccans make up 9% of the population in Amsterdam East (which is similar to their representation at the national-level), while Amsterdam New-West has a much higher population of Moroccans, with almost 20% [18]. The Amsterdam-East neighborhood was built in the 1800s and has recently become gentrified, resulting in shops, restaurants and stores that cater to its ethnically diverse population. Amsterdam New-West was more recently built, in the 1950s and 1960s. It was purpose-built as a residential area and therefore has fewer restaurants and shops, with large grocery stores, small convenience stores and snack bars.

### 2.3. Recruitment

We used a purposive sampling strategy [22] and aimed to include first and second-generation Moroccan women over the age of 18 years living in the two neighborhoods described. This age was determined as appropriate to target individuals who are primarily involved in the food procurement process, therefore adults rather than children. Based on previous research, we expected that women were most often responsible for food procurement and cooking, so we chose to focus on this group. Participants were recruited through a number of strategies based on purposive and snowball sampling. Purposive sampling targeted community centers and participants were identified by the researcher during group meetings held at the centers. Further participants were recruited via student groups at universities in Amsterdam. Finally, interviewees were asked to suggest or recommend more participants, making use of the snowball method. This method accounted for four additional participants.

Participants were recruited on a voluntary basis via email, telephone or face-to-face. Each participant was informed that they could withdraw at any point and was asked to sign a consent form at the beginning of each interview. This informed them of the purpose of the interview, the data collection process and the privacy policy.

This research was approved by the ethical evaluation committee of the Amsterdam University Medical Center—location: Academic Medical Center.

### 2.4. Study Sample

We interviewed 18 Moroccan women with an age range of 23 to 77 years (Table 1). Fifteen of the women were first-generation immigrants and three were second-generation immigrants. Nine of the interviewees lived in Amsterdam New-West, eight of the interviewees lived in Amsterdam-East and one interviewee lived in a different neighborhood of Amsterdam. Her data were included in the study as she was frequently present at the community center in Amsterdam East and conducted the majority of her food purchasing in that neighborhood. We aimed to reach data saturation which, according to Gray [21], is likely with eight to twelve interviews. This was the case in the current study; after about seven to eight interviews in each neighborhood, there were no new emerging themes, indicating thematic saturation [23].

### 2.5. Theoretical Framework and Conceptualization of the Food Environment

The theoretical framework for this research was based on the model of dietary acculturation developed by Satia et al. (2002) [2]. This model describes a broad spectrum of factors (such as demographic and cultural factors) that are important for understanding the immigrant and the context in which they live. Overall, the immigrant experiences “exposure to host culture” as well as changes in environmental and psychological factors that can lead to dietary change. The current research emphasized the potential bi-directional relationship between immigrants and the food environment, which was defined as: the supply of food within people’s daily activity space. Components of the food environment were: accessibility, availability, affordability and acceptability of traditional, host country and other food in restaurants and shops [2,24].

Accessibility was defined as ability to access available foods, including geography, convenience, transportation, opening hours, etc. Availability was defined as the presence of food items in restaurants and shops. Affordability was whether the person could pay the price of food in restaurants and shops. Acceptability was whether or not the person felt the food matched their expectations. This could be regarding quality, food handling, religious beliefs, cultural values, attitudes and taste preference. The level of acceptability is a dynamic concept. It may be influenced by the host country’s appreciation of the immigrant’s traditional food, which can impact the immigrant’s own attitude towards their traditional food. The framework gave rise to the questions that were asked during the data collection process.

### 2.6. Data Collection

Two methods were used for data collection: semi-structured interviews and a mapping activity. The semi-structured interviews were used to gain information about the participants’ perceptions of their food environment and purchasing behavior and ask probing questions [25]. The mapping activity, conducted with four participants (n = 4) who were able to complete the activity, was used to gain further insight into participant’s perceptions around geographical location and access. This technique has been frequently used in healthcare access research to provide similar information [26]. Participants were asked to draw a map of their neighborhood on a blank piece of paper, indicating their shopping route, where they procure food, both for snacking and cooking, as well as the distance of these places from their home.

The interviews made use of an interview guide that was developed based on the theoretical framework, reviewed and tested with experts in the field and adjusted following the first three interviews. The interview topic guide started broad, with questions regarding the participant’s neighborhood and what types of shops, stores and restaurants were in the area. The second part of the interview focused on the types of foods typically consumed, with a particular attention on ethnic-specific foods, and the locations where ingredients for those foods were purchased. Further questions addressed the four aspects of the food environment (availability, accessibility, acceptability and affordability). Finally, interviewees were asked to fill in (or answer verbally) a questionnaire with basic information such as place of birth, year of immigration, age.

The interviews took place between April and June 2019 and, on average, lasted approximately 30 min. They were conducted in Dutch and took place primarily in community centers. We audio recorded and subsequently transcribed all interviews. To increase internal validity, some of the transcriptions were checked by a second researcher.

Following transcription, the recordings were deleted. Personal information, such as references to street names, restaurant names or markets, was omitted from the transcripts.

Names of the participants were stored in a separate file on the Amsterdam UMC secure server. Finally, the participants were offered a small gift card to thank them for participating in the research. In order to check the content of the interviews, we conducted two follow-up interviews with key-informants within the communities; they were asked to confirm our interpretation/understanding of the points raised by the original participants.

### 2.7. Data Analysis

The interviews were analyzed through a thematic coding process. The codebook was developed from the conceptual framework, but the process of coding and data analysis was iterative in order to ensure that emerging issues were also included. In order to increase internal validity, a second researcher coded two interviews and the results were compared [21].

## 3. Results

The findings of this study were consistent with our expectation that there is a bi-directional interaction between the immigrant group (Moroccan people in two Amsterdam neighborhoods) and their food environments. The results were consistent across the participants, regardless of the neighborhood in which they lived. Participants reported experiencing a shift during their time in the Netherlands, with an increased availability of traditional foods over that period. Furthermore, the results indicate a nuanced food procurement decision-making process. The interviewees reported balancing environmental and cultural factors in their decision-making for food procurement with an on-going process of dietary acculturation.

### 3.1. Hierarchy of Decision-Making

**Figure 1:**
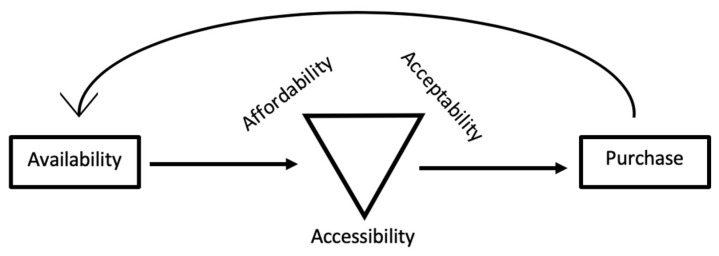
Hierarchy of decision-making and feedback loop in the food environment.

We observed a clear hierarchy in the food procurement decision-making process throughout the interviews. In order to explain this interaction, we developed Figure 1.

The “conditional” factor was availability from the acculturation perspective. First, it was necessary for ethnic-specific or traditional food items to be available, before the participants were able to consider acceptability, affordability and accessibility in their procurement decision-making. The importance of each factor was dependent on participant’s personal situation which was derived from their socio-demographic factors, migration context and cultural factors. Over time, the purchase decisions of the immigrant group presumably fed back to their food environment, thereby resulting in continued availability of the item in the food environment.

### 3.2. Availability

The interviewees reported that most traditional foods, culturally appropriate foods and host-country foods for cooking purposes were readily available for procurement in their food environment, regardless of neighborhood.

“I find everything here. I want couscous, I just go to the [big shopping street] or the [market], vegetables, get everything. The chicken or meat at the butcher and then luckily. This is our house, we have everything! Moroccan food or so.” (Interview 4, East)

Beyond the general idea that “everything” is available in both neighborhoods in Amsterdam, the food procurement practices were further divided according to five distinct food categories: meat, fish, bread, fruits/vegetables, and “other” such as spices, dairy products, sauces, etc.

Depending on the category, there were some additional acceptability requirements before the food was considered available to the women. For example, in order to adhere to religious values, meat needs to be Halal, and almost all of the participants reported eating strictly Halal. Many of the women said that they had to be able to “trust” that the meat was truly halal and one woman went as far as requesting to see a certificate as proof. If a Halal option was not available, for example, when eating out, the women reported choosing other items, e.g., fish or vegetarian dishes. Therefore, meat was considered to be “unavailable”.

Another category identified was that of “other items” (dry foods, spices, dairy). These items were typically purchased at the grocery store; however, some traditional foods such as couscous and dates were bought at the Moroccan butcher. Some of the participants perceived a change in the availability of these items over time, especially the availability within Dutch supermarkets. For example, one participant said that now it is possible to find all the spices, but that it was more difficult in the past.

“Before, for the spices, it was a bit difficult. However, now you can buy everything, even in the [Dutch supermarket] or at the [open air] market.” (Interview 12, New-West)

Despite the different locations for the procurement of foods in each category, both traditional and host-country foods were reported to be readily available within the food environment in both Amsterdam neighborhoods.

### 3.3. Balancing Acceptability, Accessibility and Affordability

Once the availability and certain aspects of acceptability, such as culturally appropriate food choices, were met, the interviewees identified several other factors that played a role in their decision to procure food. The factors were a mix of accessibility, such as convenience and location; acceptability, such as quality, shelf-life, variety, organic and freshness; affordability, meaning the price.

Interviewees identified different ways in which they weighed the various factors in their decision to procure food. The weight of each factor was dependent on the personal situation of the interviewee. The context for the personal situation can be described by the demographic and socioeconomic factors.

#### 3.3.1. Quality and Price

The first topic was balancing between quality (acceptability) and price (affordability). Many participants described struggling to find the best quality food at the lowest price. However, the responses were not consistent across interviews. For example, there were differing opinions about whether the fruits and vegetables were cheaper, better quality and safer at the open-air market or the supermarket.

“The market only if I need something quickly, you know. However, at the supermarket it is, look, the supermarket also has controls and that is truly safe. Yeah in the market, you don’t know where it came from, all the vegetables and fruit.” (Interview 15, East)

In one extreme case, price was the only factor considered. The interviewee had an extremely low income and very little money to spend on food. This led to food procurement based on the least-expensive option.

#### 3.3.2. Time and Convenience

Interviewees also weighed time (accessibility) and convenience (accessibility) in their food procurement practices. Many spoke about how this evolved throughout their life, suggesting that at different phases, time and convenience received different weights. One woman, who was retired and had free access to public transportation, traveled far to find items at a lower price, but admitted this was impossible for younger women with children.

“Time is really important for mothers to do the grocery shopping. Really important. Before, I didn’t have any time, I could not concentrate on what I was going to eat, what I was going to buy. I did, what was easy, what was fast, what was close by. However, now, with time, I concentrate on it.” (Interview 17, East)

Overall, the evolving personal situation throughout their lifetime influenced interviewees’ procurement decision-making in different ways. While the balance between affordability, accessibility and acceptability was different for each interviewee, there was general consensus about finding the best quality for the lowest price and at the most convenient location.

#### 3.3.3. Nuanced Acceptability: Difference between Morocco and The Netherlands

One theme that emerged from the interviews was the difference in taste of food between Morocco and the Netherlands. While women found that traditional foods, as well as fruits and vegetables, were readily available and acceptable in the Netherlands, when asked about differences with Morocco, their reactions were notable. This indicates a potentially more nuanced version of acceptability.

Many talked about food from Morocco with an observed nostalgia. They were reluctant to criticize the Netherlands, but when they began talking about the food in Morocco, they often mentioned a “lack of flavor” of produce in the Netherlands. The main differences were identified in fruits and vegetables, meat, fish and olives/olive oil with a “lack of sun” being an often cited reason for this difference. One second-generation interviewee said that she felt a “difference” in Morocco but was unable to fully describe it, she thought maybe it was related to the ambiance. The perceived difference in taste between the Netherlands and Morocco was not given as a reason to change food procurement behaviors, nor was it cited as a problem, but their evaluation of acceptability had shifted with the move and resulted in a seemingly changed “standard” for acceptability.

### 3.4. Balancing Other Factors to Procure Traditional or Host-Country Foods

Within the process of dietary acculturation, environmental factors play an important role in food procurement and this study focused primarily on those aspects. It was reported that the environmental factors were “satisfied”, with both traditional and host-country foods being readily available, accessible, acceptable and affordable. However, there were other reasons given for shifting from traditional foods to host-country foods that could also be attributed to the food environment.

#### 3.4.1. Family Member Preferences and Fast Food

One of the most-cited reasons for eating either host country or traditional foods were the preferences of children and husbands. Children often wanted to eat “Dutch” foods, reported by the interviewees to be dishes, such as pizza, pasta and lasagna. This was often related to what they had observed in their environment, for example, what other kids at school ate. Several of the women also stated that they needed to find a balance with their husband’s preference for traditional foods. One interviewee explained that her husband did not like Lasagna, but that her children loved it. In the beginning, when she made Lasagna for her children, she also prepared a traditional dish for her husband, but recently she decided that it was too much work and that her husband should adapt.

“In the last period of time, I said [to my husband], now that is your food.” (Interview 11, New-West)

Another interviewee reported that she loved her children, and if her children were happy, she was happy. She therefore made more host-country food to suit the children’s preferences. Later in the interview, she said that her children liked to eat Moroccan bread at home but wanted to have “sliced bread” (typical for Dutch culture) when they went to school. She said that they wanted to be like the other Dutch kids.

The children’s behavior was also reported to influence the frequency of eating out. Children’s desire to eat fast food resulted in many participants eating more fast food, which was also reported as readily available and accessible in the food environment. In the decision to acquiesce to the children’s preferences, the child’s happiness was most often given as the reason. However, women reported being concerned about the frequency of their children eating out or buying snacks on their own because they viewed this as unhealthy. This was discussed in follow-up interviews with our key figures. Both interviewees confirmed that they noticed many young people eating out. One of the interviewees described how her conversation with her young adult son changed her opinion. As a Muslim, her son said he did not drink, smoke or party. Going out to eat was his “only fun thing”. This shifted her opinion and reduced her worries about young people eating out.

#### 3.4.2. Healthy Eating

Healthy eating was another reason cited for eating host-country foods in preference to Moroccan food. Several of the interviewees reported being vegetarian, or that they did not like to eat too much meat. They reported that Moroccan dishes often contained a lot of meat and it was therefore easier to cook some vegetables with rice, potatoes or fish instead. There was a perception that Moroccan food was “fatty” or would cause weight gain. One interviewee said that Moroccan food was meant to be eaten with bread, but as she was avoiding bread for health reasons, she did not want to eat Moroccan dishes. Other dishes, such as sushi, fish and salads, were reported to be “healthy”. Overall, about half of the interviewees referred to health as driver for the procurement and preparation of various types of food.

## 4. Discussion

The objective of this study was to explore the relationship between the food environment and food procurement behaviors in the process of dietary acculturation in people of Moroccan-origin living in Amsterdam. This study found that women of Moroccan origin in Amsterdam are balancing many factors in their food procurement decision-making. First of all, participants reported that originally there was little acceptable traditional food available but that there was an improvement over time, indicating that immigrant groups have influenced the food environment. Considering availability as a principal requirement, we found that immigrants then balance other factors such as quality, price and convenience in food procurement of both traditional and host-country foods. In general, the best quality and most convenient food for the lowest price was selected without specific regard for the origin of the foods. The decision between host-country and traditional food was then made through an additional decision-making process. This study found that family member preferences, including children’s preferences for fast food, and health were most influential in this process.

The perceived availability of traditional Moroccan food items in both neighborhoods in Amsterdam indicates a change in the food environment. This was confirmed by some of the women with a longer residence duration; they reported certain spices or traditional foods becoming easier to find. This finding is an important refinement of our understanding of the process of dietary acculturation. It appears that not only does the food environment influence the procurement behaviors of the immigrant group, but the immigrant group can also cause a shift in the food environment. The model of dietary acculturation from Satia et al. [2] does not discuss this potential interaction. Holdsworth et al. [24] proposed a framework based on systems thinking, acknowledging the complex relationship between all the different factors affecting dietary and physical activity behavior. The current study expands this proposition by attempting to untangle the interaction between different determinants of diet and dietary acculturation and adds that the immigrant group itself be considered as one of the influencing factors.

The results also indicate the importance of considering the complex nature of the decision-making process and providing options that suit the different needs of the individual. This is also consistent with the model developed by Holdsworth et al. [24], which stipulated that the factors in dietary decision-making have a complex, interactive relationship. Similarly, Mattioni et al. [27] used a sociological approach to the food environment, which also explored the complex nature of the interaction between food procurement and food retail, albeit outside the context of dietary acculturation. Holdsworth et al. [24] concluded that food price had the biggest potential impact on behavior, while Mattioni et al. [27] identified quality as the driver for individual’s food procurement behavior. Interestingly, a study by Mackenbach et al. [28] found that Moroccan people in the Netherlands managed to eat a healthier diet at a lower cost than Dutch participants. This might reflect the availability of culturally acceptable foods in Amsterdam as was indicated in the current study which also indicates that Moroccan women consciously weigh up quality, health and price when purchasing food. Thus, while price (affordability) may be a determining factor in dietary behavior in general and acculturation in particular, it indicates the necessity to study individual groups and their interaction with the food environment.

The findings have implications for the promotion of healthy eating practices and overall health in immigrant groups. Dietary acculturation can positively or negatively affect health, but it has been found that certain changes to typical western diet, such as bigger portion size and higher fat and sugar consumption, negatively affect health [29]. Some immigrant groups also face social pressures to consume more foods within new food environments [30]. Furthermore, we know that immigrants tend to suffer higher burdens of diet-related diseases such as obesity, diabetes and cardiovascular diseases [3,4,5]. Therefore, in certain contexts, it may be necessary to facilitate the availability of traditional food items to encourage the continued consumption of a traditional diet. The research is unclear regarding the necessary proximity of retail outlets on food procurement, for example, in a study of refugees in Australia, by Pereira et al. [31] it was found that the presence of a grocery store within 1 km facilitated more consumption of fruits and vegetables. Similarly, in a review, Black et al. [32] found trends for increased availability of healthy options relating to healthier diet. In addition, a longitudinal study by Bivoltsis et al. [33] showed the introduction of healthy food outlets support healthier diets in Perth, Australia. Yet, Ghosh-Dastidar et al. [34] found that building a supermarket in a food desert in the United States did not result in better dietary intake. These studies, however, are more difficult to compare within the European context, where distances are generally smaller. In Amsterdam, for example, grocery stores are ubiquitous and there are readily available ethnic-specific shops and restaurants. Thus, this should be further studied within a European context. From a broad public health perspective, policy makers should ensure an economic and political environment that facilitates the availability of a variety of foods, including encouraging immigrants to participate in shaping their food environment, for example, by starting their own food businesses.

Finally, family member preferences can shift dietary behaviors and these preferences may also be influenced by the food environment. Children were reported to prefer eating host-country foods, or refusing to eat traditional foods, especially in social environments such as school. Furthermore, children were more likely to want to eat out or at fast food restaurants. While the decision to eat at fast food restaurants as compared to other restaurants was not specifically elaborated on, the concerns about affordability identified in general food procurement may be relevant here. It would therefore stand to reason that eating out options were limited to the more affordable fast food establishments. This is consistent with the findings of Osei-Kwasi et al. [35], which concluded that the availability of fast food and children’s preferences drove unhealthy eating behaviors in Great Britain. Children’s preferences in determining family eating behavior is also consistent with Mellin-Olsen et al.’s study on Pakistani immigrants in Norway [36], which found children’s preferences to be a reinforcing factor in dietary change. Greder et al. [37] also identified Latina mothers as the gatekeepers to the healthy eating of their children, but recognized that it was perceived as difficult for mothers to navigate cultural food practices and new food environments. The variation among different immigrant groups might be due to different cultural norms regarding the role of the child within the family. The present study confirms previous findings and contributes further to the implication that children can have a significant impact on the process of dietary acculturation and dietary decision-making in immigrant families. The “power” of the children in deciding the family dietary practices could have larger implications for intervention and educational programs.

### 4.1. Strengths and Limitations

There are some limitations to this study that should be acknowledged. First of all, participants were identified primarily through community centers. This limits the participant pool to people who are already active within the community and may miss an important, more marginalized group. It also limited the pool to primarily first-generation immigrants. Secondly, as previously mentioned, there was no major difference between the participants in each neighborhood. This may indicate the need to gather data from a more isolated group outside of a major city. Finally, the interviewer is also of migrant status and a non-native Dutch speaker. There are both strengths and weaknesses with this construction. Some of the participants expressed feeling more comfortable speaking with another non-native Dutch speaker. This may have helped increase rapport with the interviewees. However, the non-native linguistic skills of both interviewee and interviewer may have also limited what was able to be communicated. In a few cases, the interviewee struggled to find the word they wanted to use. This was usually solved by describing or using hand gestures. Finally, the methodological construction originally planned to ask all participants to draw a map of their food environment. It was quickly assessed that this was not possible for all interviewees. Many of the interviewees had a low education level and were not able to read or write. It was therefore not possible to carry out the mapping activity with all participants. Where the maps could be used, they served to reiterate the information given about the shops and stores in the participants’ neighborhood. The educational level of participants should be considered for future studies attempting to use this method.

### 4.2. Recommendations and Future Research

Despite the limitations of the study, the findings have significant implications for future research. For example, this study exemplifies the importance of geographically and culturally specific studies regarding dietary behavior and its potential health implications. Particularly, the universal components of food environments should be considered alongside the additional drivers of the choice for traditional foods. Further qualitative studies can give a voice to different groups to understand the mechanisms through which they interact with the food environment. Furthermore, the findings add an element of bi-directional interaction between immigrant groups and the food environment. Future studies could explore immigrant groups that have more recently arrived in a new food environment to better understand the early phase of interaction with the food environment. Immigrant groups in other settings should also be considered, for example: small populations of immigrants in an urban setting may have less impact on the food environment, or the interaction with the food environment will differ when immigrants arrive in less ethnically diverse areas, such as rural or remote towns.

## 5. Conclusions

This study contributes to a better understanding of the role of the food environment in the process of dietary acculturation. Not only does the food environment influence the immigrant group’s dietary behavior, but, over time, the immigrant group can also influence the food environment. The availability of food acts as a conditional factor in the food environment, before affordability, acceptability and accessibility can be weighed. Food environments act within a complex system but are relevant to be studied in relation to dietary acculturation in immigrant groups.

## Figures and Tables

**Table 1:** Interview participant characteristics.

Interview ID	Neighborhood	Age	Age at Migration	Years since Migration
1	New-West	68	26	42
2	New-West	52	0	52
3	East	52	19	33
4	East	54	16	38
5	East	63	40	23
6	New-West	40	23	17
7	New-West	41	24	17
8	New-West	77	40	37
9	East	65	45	20
10	New-West	44	26	18
11	New-West	44	27	17
12	New-West	63	19	44
13	New-West	26	N/A *	N/A
14	Westerpark	63	19	44
15	East	40	22	18
16	East	31	30	1
17	East	66	33	33
18	East	23	N/A	N/A

* N/A means not applicable for participants of second-generation.

## Data Availability

The data provided in this study are available on request from the author Mary Nicolaou m.nicolaou@amsterdamumc.nl. The data are not publicly available due to the privacy of the participants.

## References

[1] Dekker L.H., Snijder M.B., Beukers M.H., de Vries J.H.M., Brants H.A.M., de Boer E.J., van Dam R.M., Stronks K., Nicolaou M. (2011). A Prospective Cohort Study of Dietary Patterns of Non-Western Migrants in the Netherlands in Relation to Risk Factors for Cardiovascular Diseases: HELIUS-Dietary Patterns. BMC Public Health.

[2] Satia-Abouta J., Patterson R.E., Neuhouser M.L., Elder J. (2002). Dietary Acculturation: Applications to Nutrition Research and Dietetics. Am. Diet. Assoc..

[3] Brussaard J., van Erp-Baart M., Brants H., Hulshof K., Löwik M. (2001). Nutrition and Health among Migrants in the Netherlands. Public Health Nutr..

[4] Huisman M.J., Soedamah-Muthu S.S., Vermeulen E., Muilwijk M., Snijder M.B., Nicolaou M.N., Van Valkengoed I.G.M. (2018). Does a High Sugar High Fat Dietary Pattern Explain the Unequal Burden in Prevalence of Type 2 Diabetes in a Multi-Ethnic Population in the Netherlands? The HELIUS Study. Nutrients.

[5] Perini W., Snijder M.B., Peters R.J.G., Stronks K., Kunst A.E. (2018). Increased Cardiovascular Disease Risk in International Migrants Is Independent of Residence Duration or Cultural Orientation: The HELIUS Study. J. Epidemiol. Community Health.

[6] Swinburn B., Sacks G., Vandevijvere S., Kumanyika S., Lobstein T., Neal B., Barquera S., Friel S., Hawkes C., Kelly B. (2013). INFORMAS (International Network for Food and Obesity/Non-Communicable Diseases Research, Monitoring and Action Support): Overview and Key Principles. Obes. Rev..

[7] Zenk S.N., Schulz A.J., Kannan S., Lachance L.L., Mentz G., Ridella W. (2012). Neighborhood Retail Food Environment and Fruit and Vegetable Intake in a Multiethnic Urban Population. Am. J. Health Promot..

[8] Harrington D., Hurley K.M., Cockerham A., Black M.M., Hager E.R., Harding J., O’Reilly N. (2016). Food Swamps and Food Deserts in Baltimore City, MD, USA: Associations with Dietary Behaviours among Urban Adolescent Girls. Public Health Nutr..

[9] Mclerney M., Csizmadi I., Friedenreich C.M., Uribe F.A., Nettel-Aguirre A., McLaren L., Potestio M., Sandalack B., McCormack G.R. (2016). Associations between the Neighbourhood Food Environment, Neighbourhood Socioeconomic Status, and Diet Quality: An Observational Study. BMC Public Health.

[10] Terragni L., Garnweidner L.M., Pettersen K.S., Mosdøl A. (2014). Migration as a Turning Point in Food Habits: The Early Phase of Dietary Acculturation among Women from South Asian, African, and Middle Eastern Countries Living in Norway. Ecol. Food Nutr..

[11] Lake A., Townshend T. (2006). Obesogenic Environments: Exploring the Built and Food Environments. J. R. Soc. Promot. Health.

[12] Delavari M., Sønderlund A.L., Mellor D., Mohebbi M., Swinburn B. (2014). Exploring Obesogenic Environments: The Design and Development of the Migrant Obesogenic Perception of the Environment Questionnaire (MOPE-Q) Using a Sample of Iranian Migrants in Australia. BMC Public Health.

[13] Franzen L., Smith C. (2010). Food System Access, Shopping Behavior, and Influences on Purchasing Groceries in Adult Hmong Living in Minnesota. Am. J. Health Promot..

[14] Osei-Kwasi H.A., Boateng D., Danquah I., Holdsworth M., Mejean C., Terragni L., Powell K., Schulze M.B., Owusu-Dabo E., Meeks K. (2019). Acculturation and Food Intake Among Ghanaian Migrants in Europe: Findings From the RODAM Study. J. Nutr. Educ. Behav..

[15] Rodriguez P.I., Dean J., Kirkpatrick S., Berbary L., Scott S. (2016). Exploring Experiences of the Food Environment among Immigrants Living in the Region of Waterloo, Ontario. Can. J. Public Health.

[16] Azghari Y., Hooghiemstra E., van de Vijver F. (2017). Historical and Social-Cultural Context of Acculturation of Moroccan-Dutch. Online Read. Psychol. Cult..

[17] CBS CBS Immigration Report 2020. https://longreads.cbs.nl/integratie-2020/bevolking/.

[18] Onderzoek, Informatie En Statistiek (OIS). https://www.ois.amsterdam.nl/feiten-en-cijfers/amsterdam/?20050.

[19] Snijder M.B., Galenkamp H., Prins M., Derks E.M., Peters R.J.G., Zwinderman A.H., Stronks K. (2017). Cohort Profile: The Healthy Life in an Urban Setting (HELIUS) Study in Amsterdam, the Netherlands. BMJ Open.

[20] Perini W., van Valkengoed I.G.M., Snijder M.B., Peters R.J.G., Kunst A.E. (2020). The Contribution of Obesity to the Population Burden of High Metabolic Cardiovascular Risk among Different Ethnic Groups. The HELIUS Study. Eur. J. Public Health.

[21] Gray D.E., Seaman J. (2014). Doing Research in the Real World.

[22] Palinkas L.A., Horwitz S.M., Green C.A., Wisdom J.P., Duan N., Hoagwood K., Angeles L., Northwest K.P. (2015). Purposeful Sampling for Qualitative Data Collection and Analysis in Mixed Method Implementation Research. Dent. Surv..

[23] Saunders B., Sim J., Kingstone T., Baker S., Waterfield J., Bartlam B., Burroughs H., Jinks C. (2018). Saturation in Qualitative Research: Exploring Its Conceptualization and Operationalization. Qual. Quant..

[24] Holdsworth M., Nicolaou M., Langøien L.J., Osei-Kwasi H.A., Chastin S.F.M., Stok F.M., Capranica L., Lien N., Terragni L., Monsivais P. (2017). Developing a Systems-Based Framework of the Factors Influencing Dietary and Physical Activity Behaviours in Ethnic Minority Populations Living in Europe—A DEDIPAC Study. Int. J. Behav. Nutr. Phys. Act..

[25] Tong A., Sainsbury P., Craig J. (2007). Consolidated Criteria for Reporting Qualitative Research (COREQ): A 32-Item Checklist for Interviews and Focus Groups. Int. J. Qual. Health Care.

[26] Ensign J., Gittelsohn J. (1998). Health and Access to Care: Perspectives of Homeless Youth in Baltimore City, USA. Soc. Sci. Med..

[27] Mattioni D., Loconto A.M., Brunori G. (2020). Healthy Diets and the Retail Food Environment: A Sociological Approach. Health Place.

[28] Mackenbach J.D., Dijkstra S.C., Beulens J.W.J., Seidell J.C., Snijder M.B., Stronks K., Monsivais P., Nicolaou M. (2019). Socioeconomic and Ethnic Differences in the Relation between Dietary Costs and Dietary Quality: The HELIUS Study. Nutr. J..

[29] Popovic-Lipovac A., Strasser B. (2015). A Review on Changes in Food Habits Among Immigrant Women and Implications for Health. J. Immigr. Minor. Health.

[30] Nicolaou M., Doak C.M., van Dam R.M., Brug J., Stronks K., Seidell J.C. (2009). Cultural and Social Influences on Food Consumption in Dutch Residents of Turkish and Moroccan Origin: A Qualitative Study. J. Nutr. Educ. Behav..

[31] Pereira C.A.N., Larder N., Somerset S. (2010). Food Acquisition Habits in a Group of African Refugees Recently Settled in Australia. Health Place.

[32] Black C., Moon G., Baird J. (2014). Dietary Inequalities: What Is the Evidence for the Effect of the Neighbourhood Food Environment?. Health Place.

[33] Bivoltsis A., Trapp G., Knuiman M., Hooper P., Ambrosini G.L. (2020). Do Changes in the Local Food Environment within New Residential Developments Influence the Diets of Residents? Longitudinal Results from RESIDE. Int. J. Environ. Res. Public Health.

[34] Ghosh-Dastidar M., Hunter G., Collins R.L., Zenk S.N., Cummins S., Beckman R., Nugroho A.K., Sloan J.C., Wagner L., Dubowitz T. (2017). Does Opening a Supermarket in a Food Desert Change the Food Environment?. Health Place.

[35] Osei-Kwasi H.A., Powell K., Nicolaou M., Holdsworth M. (2017). The Influence of Migration on Dietary Practices of Ghanaians Living in the United Kingdom: A Qualitative Study. Ann. Hum. Biol..

[36] Mellin-Olsen T., Wandel M. (2005). Changes in Food Habits among Pakistani Immigrant Women in Oslo, Norway. Ethn. Health.

[37] Greder K., de Slowing F.R., Doudna K. (2012). Latina Immigrant Mothers: Negotiating New Food Environments to Preserve Cultural Food Practices and Healthy Child Eating. Fam. Consum. Sci. Res. J..

